# Assessing the Safety Risks of Civil Engineering Laboratories Based on Lab Criticity Index: A Case Study in Jiangsu Province

**DOI:** 10.3390/ijerph17176244

**Published:** 2020-08-27

**Authors:** Yi Zhang, Peng Mao, Hongyang Li, Yuxin Xu, Dan You, Hui Liu, Wei Huang, Jingfeng Yuan

**Affiliations:** 1 Department of Construction Management, College of Civil Engineering, Nanjing Forestry University, Nanjing 210037, China; zhangyi@njfu.edu.cn (Y.Z.); xuyuxin@njfu.edu.cn (Y.X.); youdan@njfu.edu.cn (D.Y.); liuhui@njfu.edu.cn (H.L.); 2 Business School, Hohai University, Nanjing 211100, China; lihy@hhu.edu.cn; 3 School of Civil Engineering and Transportation, South China University of Technology, Guangzhou 510641, China; 4 State Key Laboratory of Subtropical Building Science, South China University of Technology, Guangzhou 510641, China; 5 School of Civil Engineering, Sanjiang University, Nanjing 210012, China; huang_wei@sju.edu.cn; 6 Department of Construction and Real Estate, School of Civil Engineering, Southeast University, Nanjing 210096, China; jingfeng-yuan@seu.edu.cn

**Keywords:** civil engineering laboratories, safety risk assessment, LCI, risk factors, worsening factors

## Abstract

With the rapid development of the construction industry, an increasing amount of attention were paid by universities to the development of civil engineering experiment courses so as to improve the practical research abilities of students. In recent years, due to the frequent occurrence of civil engineering laboratory accidents, it has become an urgent issue regarding on what factors influencing safety risks and how to assess and reduce the safety risks in civil engineering laboratories. Based on the lab criticity index (LCI) model, the research specificities of civil engineering laboratories were analyzed through literature review and expert interviews and 13 risk factors of civil engineering laboratories, from the four aspects of man, object, management, and environment, identified. The data for each parameter in the LCI model was obtained through a questionnaire survey, and finally the LCI value was calculated to evaluate priority. Among them, insufficient safety awareness of operators, danger due to equipment failure, imperfect management policies, and complex floor conditions were listed as the most common risk factors. Based on the LCI model, the worsening factors of these four risk factors were further analyzed. The LCI model is applied to the new research field of safety risk assessment in civil engineering laboratories that few researchers have studied before and a risk list for civil engineering laboratories was created. We revealed the safety status of civil engineering laboratories in Jiangsu Province and provided feasible suggestions for improving the management and supervision of civil engineering laboratories at universities. It can strengthen operator awareness of the risks in civil engineering laboratories and improve the social group’s attention to the safety risks of the laboratories, thus reducing the accidents’ possibility and seriousness of civil engineering laboratories.

## 1. Introduction

A university laboratory is a serious workplace that needs to be studied [1], and it is expected to be the main place for the implementation of inquiry practice [2]. Therefore, its safety must be prioritized [3]. However, students often ignore the importance of laboratory safety when conducting experiments. If there is no proper safety management procedure, the university laboratories will be in danger at any time. Although there are many discussion about university laboratories at this stage, the safety risks of university laboratories remains poor developed, as compared to the safety practice generally emphasized in industrial laboratories [4]. Therefore, laboratory safety should be an important issue in all experimental courses in universities.

According to incomplete statistics, 112 typical accidents occurred in university laboratories in China from 1999 to 2016, resulting in 12 deaths and 84 injuries [5]. From 2000 to 2015, there were 34 laboratory accidents and 49 deaths in the United States, 11 of which occurred in internal university laboratories [6]. Therefore, universities need to better manage the safety of laboratories to ensure the safety of students, teachers, and other operators.

Although most of the existing studies involve laboratory safety risks, experts and scholars all over the world pay little attention to civil engineering laboratories. Compared to other types of laboratories, civil engineering laboratories have more experiments on the mechanics and large-scale equipment. The risk coefficient of experimental equipment is therefore great, and the requirements and abilities for the regular maintenance of equipment are high [7]. Due to the dense distribution of equipment and the variety and complexity of experiments, collision and safety accidents are more likely to occur during experiments. In recent years, safety accidents in civil engineering laboratories occurred frequently and had serious consequences. On 19 May 2015, a 25-year-old graduate student in the laboratory of the Civil Engineering Department at Taiwan University cut his hand, face, and chest with broken glass due to careless operation of concrete pressure equipment. On 26 December 2018, the Municipal and Environmental Engineering Laboratory of the School of Civil Engineering and Architecture of Beijing Jiaotong University was burned and exploded, resulting in the death of three students [8]. Therefore, the safety of the civil engineering laboratories needs to be studied urgently.

However, at present, the researches on the risk of civil engineering laboratories mainly focused on the laboratory construction [9] and teaching reform [10]. Few scholars paid attention to the risk and conduct research on the safety risk. In much discussion of university laboratories, most of them have no specific distinction or pertinence. In the research on specific laboratory safety risks, there are few civil engineering laboratories involved, and the studies lack assessments and analyses of civil engineering laboratory safety risks.

To fill the research gap mentioned above, three specific objective were following proposed: (1) how to assess the safety risks of new field of civil engineering laboratories that few researchers had studied before and whether the LCI model (suggested by Ouedraogo et al. [11,12] to assess risks for research/academia environment) is suitable for civil engineering laboratories, (2) what factors influencing the safety of civil engineering laboratories and (3) what is the risk status of civil engineering laboratories in Jiangsu Province and how to reduce the safety risks in civil engineering laboratories. Through the research of these purposes, a theoretical basis for the management of the civil engineering laboratories in universities can be provided to improve the management and supervision system. The personal safety at the laboratories will be further ensured and the possibility and severity of civil engineering laboratory accidents are expected to be reduced.

## 2. Literature Review

The current laboratory safety management measures mainly focus on improving safety culture and management methods. Concerning management measures to eliminate laboratory safety problems, Johnson et al. [13] believed that laboratory safety practices and safety culture needed to be improved. He thought the importance of laboratory leadership needed to be emphasized. Leadership is the key to establish a strong safety culture. Strong safety leadership can improve safety performance and reduce the occurrence of laboratory accidents [14,15,16]. In addition, some researchers have dig into management methods. Some scholars supported laboratory hazard identification and risk management by implementing a chemical health risk assessment project, bowtie method, or developing a laboratory at-risk behavior and improvement system [3,17,18]. Some communication channels were established to share laboratory procedures among different laboratories and a more open platform for scientists to discuss laboratory safety was provided [19].

In research related to the risk factors of laboratories, some scholars have studied human unsafe behaviors. They found that the most common factors leading to laboratory accidents were man-made operation errors. If operators violate the safe operating procedures in the laboratory, they will cause injuries or even death [3,20,21]. Another group of scholars studied the unsafe state of objects, such as the electrical hazards of national laboratory equipment [22]. There are also some studies on management factors. They pointed out that laboratories were homes to dangerous goods, precision machinery, and equipment. If the dangerous goods or precision machinery are not handled properly or there is a lack of fire safety measures, the risks of the laboratory will be increased and serious injury caused [23]. Besides, the laboratory environment has been studied, e.g., the noise level in the laboratory [24]. Some scholars also combined multiple risk factors and thought that management factors and personal factors will have influence on the safety environment of university laboratories [25,26,27]. Stroud et al. [28] found that poor ventilation and lack of knowledge of laboratory safety management were often ignored. It can be therefore seen that, currently, the researches on risk factors in laboratories are rare and scattered, and there is no systematic and comprehensive research on risk factors.

The existing research on the safety risks in university laboratories mostly concentrate on chemical laboratories [16,17], and there is little research on civil engineering laboratories. There are some existing studies on the civil engineering laboratories in universities, but mainly focusing on the laboratory construction [9], teaching reform [10,29], etc., while rarely on the risk issues.

To sum up the research on laboratory safety risks, on the one hand, the existing literatures focus on improving safety culture and management methods, and there is no systematic and complete identification of risk factors. On the other hand, most existing researches on laboratory safety risks are focused on other types of laboratories, and there is relatively little research on civil engineering laboratory risks.

## 3. Identification of Risk Factors and Research Specificities

Civil engineering laboratories generally include soil mechanics laboratories, shaking table laboratories, impact force laboratories, and concrete preparation and maintenance laboratories, etc. [30]. Among them, there are many large-scale machines and no protection device outside of the equipment. If the experimenter is not concentrating, the mechanical injury may occur. If the mechanical equipment is not rigid enough and has poor stability, it may cause hit by objects, falling objects injuries, and so on.

Like chemical laboratories, civil engineering laboratories have chemicals for students majoring in water supply and drainage and building environment to use. The incorrect use of these chemicals can cause poisoning and suffocation. If corrosive chemicals are not used properly, such as acids and alkaline metals, they will cause burns [31]. Compared to general laboratories, civil engineering laboratories may also explode due to improper use of gas cylinders or problems of gas cylinders themselves, and the flammable sundries in laboratories may also cause fire, etc. When mechanical equipment is used in civil engineering related experiments, electricity will be involved. If it is used improperly or overloaded, there can be electric shocks, leakages, static electricity, and other electrical hazards. In addition, anything that touches a high-temperature furnace or heating equipment will be burned.

After fully understanding the risk of a civil engineering laboratory, its potential risk factors were deeply studied. Based on Classification and Code of Hazardous and Harmful Factors in Production Process (GB1386-2009) and the 4M1E analysis method [32] (4M refers to man, machine, material, method, and 1E refers to the environment), on-site investigation and consulting relevant literature, and through full communication with experienced experts, the risk factors of civil engineering laboratories were divided into four categories: human unsafe behaviors (Man), the unsafe state of objects (Object), management factors (Management), and laboratory environment (Environment). Table 1 summarizes the detailed classifications.

The human unsafe behaviors refers to the operators’ lack of ability [3,20,33], safety awareness [34,35,36], and poor physical and psychological conditions [37,38]. For example, during the installation of building water supply and drainage system pipes and the measurement and calculation of fire hydrant water supply systems, laboratory personnel should not play in the experimental site or touch the power supply casually, avoiding personal injury caused by falling into the pool or electric shock accidents.

The unsafe state of objects refers to the danger of the test product or the mechanical equipment itself [22,23], and the danger of the equipment due to the failure [23,39,40,41]. For example, flammable, toxic, and corrosive reagents are often used in chemical experiments of building environment and water supply and drainage specialties. In the load compression mechanical test, the objects may be broken. There are many analytical instruments in the laboratory for environment specialty that need to be heated to a very high temperature during the experiment, such as digesters, which need to be cut off when not in use. If the instruments are old and the power supply is not cut off in time, accidents are likely to happen.

Management factors refer to imperfect management policies [18,28,35], capital risk [42,43], and poor execution of safety management [44]. For example, the investment of laboratory protective equipment is insufficient and the fire alarm facilities are imperfect.

The laboratory environment refers to the bad environment that may exist in the civil engineering laboratories, including strong noise vibration [24,45], adverse temperature and humidity [46,47], bad ventilation [28,48,49,50], poor lighting [51,52] and complex floor conditions [35,41,53]. For example, the electro-hydraulic servo universal testing machine requires that the noise value of laboratory environment should be at least 10 dB (A) lower than the weighted sound level of machine. Temperature and humidity are important factors affecting the performance of instruments. They can cause corrosion of mechanical parts in the civil engineering laboratories, and cause errors or performance degradation of mechanical parts of instruments. 

Civil engineering laboratories, as a kind of academic laboratories in universities, are unique, especially when comparing to common industrial laboratories. The pressure related to safety of doing experiments in university laboratories is different from that of industrial laboratories, and it is impossible to have the same level of understanding of the issue [54]. In the current laboratory-related research, industrial laboratories were found to more emphasize the safety of employees while the safety management in the academic laboratory in universities is not mature [41,55], not to mention civil engineering laboratories. The work of the industrial laboratories is a standardized operation process while the experiments conducted by the students in academic laboratories, and the laboratory environment in which they are located frequently change. They also lack the necessary corresponding safety education and training. Misunderstandings about “low risk” and “intrinsically safer” of university laboratories still exist. Part of the reason is a lack of awareness of the hazards. It is therefore generally believed that the risks associated with academic studies are far lower than those associated with large-scale process industry operations [6].

The purpose of research specificities is to differentiate between civil engineering laboratories and industrial laboratories. Groso et al. [56] summarized the possible research specificities of the academic experimental environment. According to the actual situation of civil engineering laboratories, opinions were solicited through on-site investigation of five experts with over 10 years of working experience in civil engineering laboratories and selected seven research specificities eventually. Some modifications were made according to the opinions of experts and the specific situation of civil engineering laboratories in universities, as shown in Table 2 [56].

## 4. Research Methods

In this paper, the LCI model is used to evaluate the risk factors of the civil engineering laboratories. The relevant data involved in the model was obtained from experienced teachers or laboratory staff through a questionnaire survey.

### 4.1. Lab Criticity Index

Lab criticity index (LCI) is suggested by Ouédraogo et al. [11,12] based on risk priority number (RPN) and analytical hierarchy process (AHP) for evaluating risks in research/academia environment. There are many risk assessment methods, such as hazard and operability analysis (HAZOP), fault tree analysis (FTA), event tree analysis (ETA), RPN, risk matrix, failure mode and effects analysis (FMEA), etc., but they have some defects and are not very suitable for the research of assessing the safety risks in civil engineering laboratories. Civil engineering laboratories have their own characteristics, which contain many different types of experiments involving geotechnical, mechanics, and structure, etc. Each experiment has its own unique operating procedures. As mentioned above, there are also many risk factors that affect the safety of civil engineering laboratories, including four aspects: man, object, management, and environment, which make risk assessment more complicated.

HAZOP is limited to specific experimental process assessments, analyzing the risks in the design and production stages, and is often used in petroleum and chemical industries [56]. Using FTA or ETA as assessment method, which is usually used in aerospace, nuclear power plants and the chemical industry [56], research must have specific initial events or a single initial risk resource. ETA can only calculate the probability of an accident and a large number of risk factors will complicate the assessment process. A significant flaw of RPN and risk matrix is that different risks may have the same risk level [57], in comparison, the conversion to the log function also effectively avoid the same priority results. Considering that the RPN scale in not continuous, indeed it has many “holes” [58,59], LCI is used in that the distribution of values calculated by LCI is continuous. FMEA is often used in analyzing the failure of equipment, however without involving the influence of people and comprehensive risk factors which are considered in LCI [56]. Moreover, the LCI model has been verified in the laser laboratory [12], and it can well screen out the key risk factors. One of LCI’s main characteristics was to consider research specificities and the interaction between different risk factors [60]. Therefore, this paper has conducted research based on the LCI model.

LCI is an index in order to make a better comparison to get the key risk factors. In this paper, the index system from four dimensions which has 13 risk factors is defined for civil engineering laboratories. After calculating the LCI of 13 risk factors, the data and selected risk factors with high LCI scores were compared and analyzed. The higher the LCI value is, the higher its priority should be. Aiming at the factors with higher risk priority, the internal parameters were analyzed and the parameters with the higher internal score were taken as the key point of risk management.

As for the formula (1), I_h_ is formed by worsening factors (WF), severity (S), and correction constant α. The correction constant α = 1 is to prevent the molecule becoming 0 without worsening factors, as shown in Table 3. The worsening factors present the influence among risk factors, in particular situations in which a risk can be worsened or enabled by the presence of another risk [60]. For example, in the shaking table test, when the mass block installed on the component is not bound firmly, leading to that the mass block is in an unsafe state. If the operator fails to take corresponding protective measures due to a lack of safety awareness, the mass block is likely to be broken and hurts people or causes other equipment damage. The value range of WF and D is [2,10,11] with the formula $\sum_{i=1}^{n}{\mathrm{wf}}_{i}=10$.

(1) $$I_{h}\;=\;\left[\left(\overset{m}{\underset{j=1}{\sum }}{\mathrm{wf}}_{j}÷m÷\overset{n}{\underset{i=1}{\sum }}{\mathrm{wf}}_{i}\right)+\alpha \right]\times S$$

Formula (2), for the research specificities (RS), and the total score of the original data is 35 (the maximum value of the Likert scale is 5 × 7 rs_i_), the score of rs_i_ equals original data/3.5, as shown in Table 3. The scores were added to obtain the final RS.

(2) $$\mathrm{RS}=\overset{n}{\underset{i=1}{\sum }}{\mathrm{rs}}_{i}$$

Formula (3), for hazard detectability (HD), includes availability (A), reliability (R), and selectivity (Se), as shown in Table 3. HD is the only special case. A score of 10 means the monitoring ability is very low, the risk coefficient is thus high. A score of 2 means the monitoring ability is very high, and the risk coefficient is low at this time.

(3) $$\mathrm{HD}=\frac{A+R+\mathrm{Se}}{n}$$

Formula (4), for the calculation of the probability (POA) of civil engineering laboratory accidents, includes hazard probability (HPt) of risk factors and frequency/duration (FD) of students’ experiments, as shown in Table 3.

(4) POA=HPt×FD

In conclusion, combined with the risk characteristics of civil engineering laboratories and literature [12], the LCI model in this paper is shown in formula (5). The weights of I_h_, RS, HD, POA come from the calculation of analytical hierarchy process (AHP).

(5) $$\mathrm{LCI}=\log_{1.66}\left(I_{h}\right)+\log_{5.78}\left(\mathrm{RS}\right)+\log_{6.06}\left(\mathrm{HD}\right)+\log_{17.24}\left(\mathrm{POA}\right)$$

### 4.2. Questionnaire Survey

Based on the LCI model, relevant data were collected from the survey. The questionnaire is divided into the following three parts: (1) Basic information about the respondents. Designed by the researchers themselves, the questionnaire includes the types (lab teachers or lecturers (who teach the courses having the requirements of experimental courses)), the professional categories (multiple choice questions) of the courses taught by the lecturers, and the working years of the lab; (2) investigation of internal parameters of the LCI model. The questionnaire was developed to understand the WF, S, A, R, Se, HPt, and FD of 13 risk factors in civil engineering laboratories. The questions include the extent to which the other three major risk factors will aggravate the severity of a certain risk resource (their average value is WF), the severity of the impact of a certain risk resource (that is, S), and the possibility that a certain risk resource may be identified (i.e., A, R, Se), the likelihood of the risk occurring (i.e., HPt), the frequency and time students spend in the laboratories (i.e., FD); (3) the research specificities of civil engineering laboratories. The differences between civil engineering laboratories and industrial laboratories were evaluated. According to the research on RS in Section 3, the corresponding problems are designed for the 7 aspects in Table 2, namely, free experiment, without normal risk recognition training, multicultural environment, etc. Based on the data obtained from the questionnaire, I_h_, RS, HD, POA can be calculated separately through Formulas (1)–(4), and then the LCI value of each risk resource can be calculated through Formula (5).

Part of the answer was measured on a five-point Likert scale. The higher the score is, the higher the risk will be. The typical five-level Likert project format is from “very disagree” (recorded as 1, set the score as 5) to “very agree” (recorded as 5, set the score as 1). The internal parameter result score is adjusted according to the model. The other part of the data is presented by matrix radio, which is divided into “no”, “weak”, “general”, “strong”, and “very strong”. The data processing is the same as before.

According to the survey, 118 valid questionnaires were finally collected through online questionnaire from the lab teachers or lecturers (who teach the courses having the requirements of experiment) of civil engineering laboratories of some typical comprehensive or science and engineering universities in Jiangsu Province. In advance, the teachers of these universities were contacted one-on-one and asked to send out link (electronic questionnaire) to their laboratory colleagues. All the teachers who responded to the questionnaire were all professional and familiar with the civil engineering laboratories. In this study, SPSS 26.0 was used to test the split-half reliability of the questionnaire. The data collected by the questionnaire (Table S1) were subsequently calculated according to the LCI model and the LCI value of 13 risk factors finally obtained.

## 5. Results

### 5.1. Reliability and Validity Test

In this study, as the Spearman–Brown coefficient of the questionnaire was 0.834 (greater than 0.8), the reliability of the questionnaire was acceptable [61]. This questionnaire was designed based on the LCI model, and the specific issues involved were all designed for factors. The selection of factors and design of the questionnaire were approved by experts, and the questions raised well reflect the risk situation of civil engineering laboratories. In addition, the referenced questionnaire used in the design of this version has been applied to the laboratory safety risk research by relevant scholars. The validity of the questionnaire has been verified [12]. Therefore, this questionnaire has high content validity.

### 5.2. Sample Characteristics

In this survey, 63.16% of participators were the lecturers and the rest were laboratory teachers, as shown in Table 4. It can be seen from the professional analysis conducted by the lecturers that almost all the specialties of civil engineering are covered by the required experimental courses, which makes this study of universal significance (Table 5). Moreover, 85.59% of teachers have 5 years or more of laboratory work experience, indicating that the respondents are relatively familiar with laboratory work and the reliability of the data obtained from the questionnaire is relatively high, as shown in Table 6.

### 5.3. LCI Calculation Results

Because there was no uniform standard for judging the risk level, the prescribed limits in the relevant literature were considered, which were widely accepted area (LCI 4), tolerable area (4 ≤ LCI < 6), and unacceptable area (LCI ≥ 6) for reference only [12]. This is not an absolute bound since the priority levels of risk factors were mainly evaluated to solve the problems. The higher the LCI is, the higher the risk. As shown in Table 7, the LCI scores calculated were all in unacceptable areas and needed to be managed. Regarding man, object, management, and environment, the highest scores are A2 (insufficient safety awareness of operators), B2 (danger due to equipment failure), C1 (imperfect management policies), and D5 (complex floor conditions)—indicating that these four were very serious and needed more attention. A formal risk management plan thus needed to be formulated.

A further study of most fundamental internal parameters shows that WF scores are relatively high for most risk factors, which needs to be given priority when implementing response measures and the scores for severity (S) are lower. Other parameters scores are relatively low, but they are within the tolerable range, far from being acceptable. If there is a risk resource, WF refers to the occurrence that another risk resource may accelerate or aggravate the degree of the risk [60]. For example, when A1 (insufficient capacity of operators) happens, the occurrence of risk will be accelerated if the objects are unsafe. That is to say, the unsafe state of objects is a worsening factor of A1, which value is obtained from the average value of three pieces of original data. The larger the value is, the more acceleration the occurrence of the risk is. Table 8 lists the value.

The purpose of RS is to differentiate between civil engineering laboratories and industrial laboratories. They differ from risk factors. They have no direct relationship with risk, but will make the environment of civil engineering laboratories more complex. The higher the score is, the greater the impact will be. Among them, rs3 refers to that operators changed frequently, with incomplete record experience on laboratory safety (3.737 points) and rs4 means that operators and partners were familiar with the possible risks of the laboratories which were scored relatively high (3.788 points) while other scores were relatively low. According to the analysis results (score of 7.295/10), it can be seen that they will also have a greater impact on laboratory safety risks, as shown in Table 9.

## 6. Discussion

### 6.1. Research Specificities

As stated above, the research specificities’ scores of the civil engineering laboratories are relatively high. The highest score is 3.788, indicating that most of the teachers agree with the importance of the experimental partners. If an unidentified risk cannot be managed, it will tend to expand into a major problem. If the operator and the experimental partner cannot recognize the risk at this time, it is quite possible to ignore the occurrence of relevant risk events. If the experimental partner understands the risk, he/she will always reminds the operator, or points out the risk factors in time when the operator is not operating properly, so that the operator will pay attention to the risk [56]. Therefore, civil engineering laboratory managers need to strengthen the popularization of the knowledge of laboratory risks to experimenters to reduce the possibility of the risks when conducting experiments or to find and correct mistakes in time to avoid potential risks when others carry out experiments [34].

The frequent replacement of operators leads to incomplete records of relevant experiment of laboratory safety, which also has a high score of 3.737. Not only for teachers’ scientific research, civil engineering laboratories in universities are also used for scientific research experiments conducted by postgraduate and undergraduate students. However, as a teaching and scientific research laboratory, the operators change frequently in batches. At the same time, the relevant personnel did not record some possible risks encountered in their experiments [56], which is not conducive to the improvement of the corresponding management measures. Therefore, for the experiment with high risk, special experimenters should be set up to assist in the experiment and record the relevant experiment safety experience [62]. For the general experiment, scholars should be encouraged to record their own relevant experience—thus improving laboratory safety management.

Though the scores for other aspects are relatively low, they however cannot be ignored. Therefore, it is suggested that some incentive measures for the operators should be provided by the laboratory managers [63]. The safety responsibility hierarchy for some complex and changeable projects can be set up and the safety responsibility can be implemented in place [64].

### 6.2. Human Unsafe Behaviors

**Figure 1:**
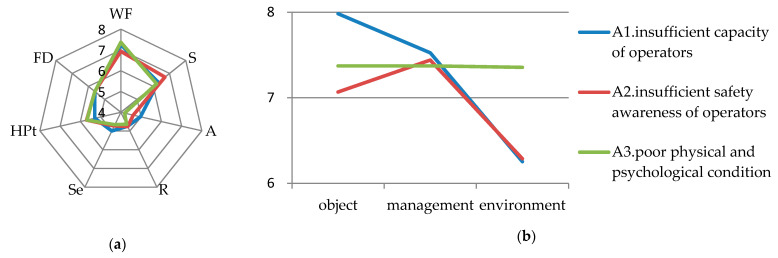
(**a**) Parameters’ scores for human unsafe behaviors; (**b**) detailed scores of worsening factors (WF) for human unsafe behaviors.

Among the risk factors of human unsafe behaviors, A2 (insufficient safety awareness of operators) has the highest LCI score of 7.097, which needs to be managed first. The scores of A1 (insufficient capacity of operator) and A3 (poor physical and psychological conditions) were relatively low (6.984 and 6.921 respectively). WF and S of these three risk factors are relatively high while the scores for the other parameters are relatively low, as shown on the left of Figure 1.

(1) As shown on the right of Figure 1, when A2 occurs, loose management will accelerate the occurrence of the risk. The operators of civil engineering laboratories in universities are mostly students who are highly mobile. Before the experiment, the teacher’s emphasis on the relevant experimental precautions will affect the operator’s cognition of the laboratory risks. However, most of the operators in industrial laboratories are highly qualified professionals who are more familiar with the experiments and experimental environment. As a result, they have a higher risk perception and a stronger safety awareness [55]. Therefore, when students ignore these precautions and teachers do not remind students about them, they become likely to engage in unsafe operation behaviors, thus resulting in increased risks [35]. Moreover, the students’ safety attitude will change after the intervention of laboratory safety education in a short time. Therefore, it is necessary to strengthen operators’ safety education and training in civil engineering laboratories to improve their safety awareness compared with industrial laboratories [34,65], which can strengthen the safety attitudes of students in the short term and improve their safety awareness during the experimental process.

Meanwhile, for A2, the score of S (severity) was higher (6.712). The reason is that man is the leader of the experiment. When man’s safety awareness is not strong, individual protection will be ignored [25], and the compliance of individual protection equipment will generally decrease with the drop of safety awareness. If protective measures are not in place, the injury of operators will be worse [55]. In addition, compared with chemical laboratories where operators are required to wear appropriate laboratory coats, operators in civil engineering laboratories do not have special clothing [66,67]. Therefore, the operators should wear appropriate individual protective equipment when entering the laboratory [68].

(2) For A1 and A3, WF had higher scores. When the operator has low ability, the probability of risks caused by the unsafe state of equipment is more likely to increase than that caused by deficiencies in man and management, as shown on the right of Figure 1. If the operator is inexperienced, she or he will not understand how to use the equipment and become unskilled in operation and insensitive to the related risks [33]. When the physiological and psychological conditions are not positive, the effects of object, management, and environment are similar. It is difficult for the operator to concentrate on the experiment and keep paying attention to the experimental process due to excessive fatigue, depression, or poor physical condition. At such a time, if there is any influence of object, management, or the environment, the deterioration of the result will be accelerated [69].

Based on the investigation, for A1 and A3, the scores of S are lower than that of A2, since the operator will be more cautious in the experimental process when he/she is inexperienced and may be assisted in the experiment. However, when the operator is excessively fatigued, with a low mood or poor physical condition, he/she usually only performs some auxiliary and risk-free tasks, such as recording data. Relatively speaking, in addition to the ability of laboratory personnel, industrial laboratories have standardized operating procedures, while the exploratory experiments in civil engineering laboratories are irregular, novel and complex [11]. Meanwhile, doing experiments is not a student’s daily routine. Therefore, students are required to keep in mind the experimental precautions before the experiment. When the experiment is more complex, the senior students should help and students can improve their experimental ability. In addition, when the operator’s physiological or psychological state is poor, it is not suitable for them to carry out complex experiments by force.

(3) For A1, A2, A3, HD (including A, R, Se) and POA (including HPt, FD), scores are relatively low, indicating that human unsafe behaviors is easy to monitor and unlikely to occur. Because in the process of the experiment, people’s unsafe behavior can be corrected in time through peer’s reminders [70], the person who can operate in the laboratory must have received certain knowledge training, and the person who does not have certain knowledge will not be allowed to experiment blindly [35]. For undergraduate students, most civil engineering experiments have well-defined operation procedures. Therefore, the possibility of accidents caused by human unsafe behaviors is relatively low.

### 6.3. Unsafe State of Objects

**Figure 2:**
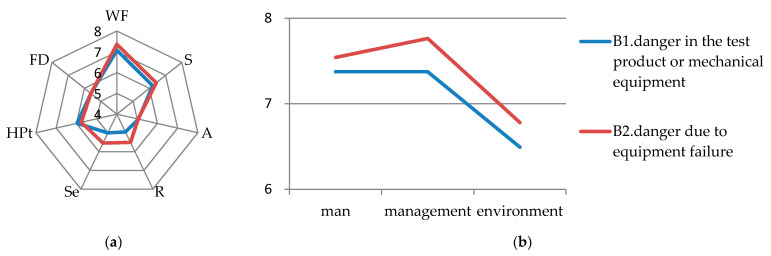
(**a**) Parameters scores for unsafe state of objects; (**b**) detailed scores of WF for unsafe state of objects.

Among the risk sources of unsafe state of objects, the LCI score of B2 (danger due to equipment failure) is the highest of 7.142, requiring priority management. The LCI score of B1 (danger in the test product or mechanical equipment) is relatively low at 7.006. For B1 and B2, the WF score is high and the S score is average, as shown on the left of Figure 2.

(1) As shown on the right of Figure 2, man and management have a great influence on the WF of B2. There is an interaction between man and object. Improper operation of the operator will aggravate the risk caused by equipment failure [71]. We found that when people’s risk awareness or abilities are not enough to detect equipment failures, accident rates will be accelerated [72]. Similarly, inadequate supervision of equipment safety and incomplete regular inspection will also increase the possibility of accidents [73]. The equipment itself is responsible for most accidents which are relevant to equipment in civil engineering laboratories. Environmental changes do not have substantial impacts on the risk—leading to the low impact of the environment on B2 [74]. Therefore, the management should regularly check the civil engineering laboratory equipment, strictly implement the instrument operation specifications, and control the risk point to the lowest level [68,73,75].

In terms of B2, the score of severity is 6.314, which is average. The use of old machinery and equipment without basic protection components by operators will indirectly lead to equipment failure [76]. Therefore, to some extent, regular equipment maintenance and supervision in civil engineering laboratories are also critical.

(2) For B1, Figure 2 shows that the impacts of man and management are not very different. The impact of the environment is the lowest among these three factors. Most of the equipment in civil engineering laboratories is relatively large. For example, the size of the general shaking table is 8 m × 10 m [77], and the size of a galvanized steel tank for model pile test is 975 mm × 695 mm × 680 mm [78]. For other instrument-related or chemical laboratories, the equipment is rarely used and the volume is much smaller. When there is danger in the experimental materials or mechanical equipment, the operator’s improper operation or the absence of warning signs will undoubtedly increase the probability of accidents [72,79]. Therefore, it is necessary to standardize the operators’ behavior and place the danger warning signs within the visible range. In the aspect of severity, according to the on-site investigation, we find that most civil engineering laboratories are confirmatory experiments, most of which operate the equipment in the form of groups with specified operating procedures. To some extent, the risk of the equipment is thus controllable.

(3) For B1 and B2, HD (including A, R, Se) and POA (including HPt and FD) have relatively low scores, as shown in Figure 2. In comparison, the HD of B2 has higher R and Se scores which means a lower ability can be identified and the risk is higher. It may be related to the failure of safety valve, leakage caused by aging and insulation of equipment, instability, unreliable power supply, etc., which are difficult to observe with the naked eye [80].

### 6.4. Management Factors

**Figure 3:**
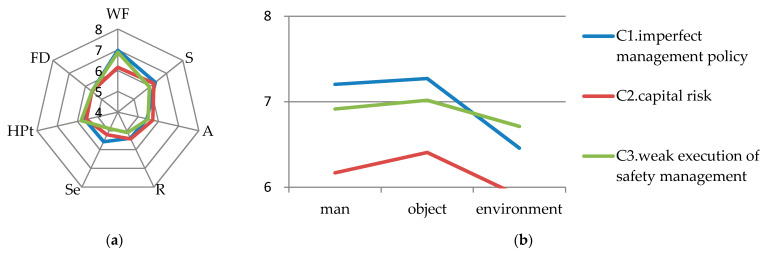
(**a**) Parameters scores for management factors; (**b**) detailed scores of WF for management factors.

Among the risk sources of management factors, the LCI score for C1 (imperfect management policies) was found to be the highest (7.047), thus requiring priority management. The LCI scores of C2 (with capital risk) and C3 (with weak execution of safety management) are relatively low, which are 6.907 and 6.855 respectively. The WF scores of C1 and C3 are higher while the scores for all parameters of C2 are not high, as shown on the left of Figure 3.

(1) As shown on the right of Figure 3, for C1, the analysis of WF shows that man and object have significant impact on C1, and their scores are relatively close. When the management policy is incomplete, the management will not consider the quality of the equipment. If the equipment is damaged at this time, it will inevitably worsen the risk. Similarly, the lack of management of students can accelerate the occurrence rate of hazards. Therefore, the management should improve the laboratory safety management system, pay attention to the status of the equipment, and strengthen the supervision of the equipment and the management of students [18,72].

(2) On the WF of C3, the impact levels of man, object, and environment were similar. If the management does not pay attention to the implementation of rules and regulations, the operators will relax the daily maintenance of machinery and equipment, as well as the pursuit of a clean environment. This will aggravate the impact of risks caused by poor management [81]. Therefore, the management should strengthen its supervision while improving the laboratory safety management system [82].

(3) Compared to C1 and C3, the scores of all parameters of C2 are not high. It can also be found that the scores of C1, C2, and C3′s S, HD, and POA are not high, which may be due to the systematic and perfect management policy formulated after various considerations, and the special department has been set up before the implementation. Therefore, it is easy to detect risk before the risk occurs, and the risk is not likely to occur due to the control of management personnel at all levels [83]. However, the management still needs to reserve sufficient funds for the construction of the laboratories and ensure that the funds are used in an appropriate manner [28].

### 6.5. Laboratory Environment

**Figure 4:**
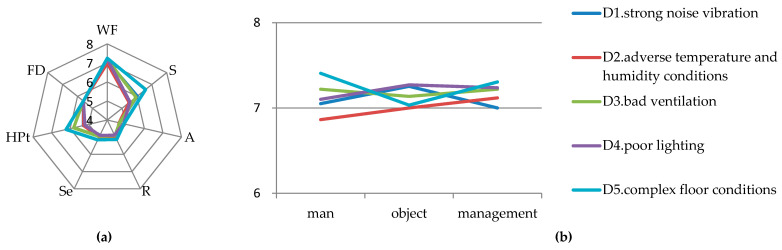
(**a**) Parameters scores for laboratory environment; (**b**) detailed scores of WF for laboratory environment.

Among the risk sources of the laboratory environment, D5 (complex floor conditions) has the highest LCI scores of 7.182, and thus needs to be prioritized. The scores of D1 (strong noise vibration), D2 (adverse temperature and humidity), D3 (bad ventilation), and D4 (poor lighting) are relatively low, which are 6.826, 6.642, 6.864, and 6.739 respectively. For D5, the scores for WF, S, and HPt are relatively high. For D1, D2, D3 and D4, the scores for WF are the highest. Meanwhile, the scores for S of D3 and D1 and the scores for HPt of D3 are relatively high, as shown on the left of Figure 4.

(1) As shown in Figure 4, for the WF of D5, when the floor conditions are complex, problems in man and management will accelerate the occurrence of risks. For civil engineering laboratories, there will be many different students doing experiments according to the needs of the course which is different in industrial laboratories where the number of operators are fixed. Meanwhile, some civil engineering laboratories do not have much space. If the operator cannot well clean the laboratory, the environment of the civil engineering laboratories will become very messy, which will affect the quality of the operator’s experiments and easily cause accidents [84,85]. Similarly, management is also an invisible driving force for a clean laboratory environment. Because the laboratory space itself is small, if there are many items on the ground but the management is poor, the ground environment will become more complex [86]. The unsafe state of the objects has slightly lower impact on it, though it cannot be ignored. Because there are many large-scale mechanical devices in civil engineering laboratories. If the ground is smooth, then the operator is prone to mechanical injury and collision. That’s why the scores for D5′s S is higher. Therefore, this kind of accident cannot be ignored, but it can be effectively reduced by maintaining a clean and tidy working ground [35]. As a result, the operator should ensure the suitability of the laboratory environment before and pay attention to the comfort of the experimenter before and after the experiment. On the one hand, operators should try their best to avoid disorderly behaviors in the laboratory, keep their laboratories clean and tidy after the completion of their experiments, and dispose of experimental waste in time [35]. On the other hand, the waste management of the laboratories should be mandatory [87]. The management of the laboratory should be regularly checked. Management should implement a reward and punishment system and punish people for disorderly placing materials. It is also necessary to publicize the adverse effects of this aspect to the teachers, take keeping the laboratories clean as a significant index for the assessment of the person in charge of each civil engineering laboratory, and let them carry out the requirements of this aspect when students do the experiment. The rewards and punishments of industrial laboratories are linked to their individual wages. Similarly, the specific reward and punishment measures of civil engineering laboratories can be considered to link with students’ usual performance. The person in charge of an industrial laboratory has absolute leadership over the staff, and it is easier to achieve job management, but this is very different from that of a university [88]. The person in charge of each civil engineering laboratory is fixed who have no direct relationship with the students, while the lab teachers are mobile and they have a direct teaching relationship with the students. Thus, it is necessary for laboratory leaders to participate in the students’ usual performance evaluation. Moreover, when the laboratory is constructed, the floor needs to be antiskid [41]. Compared to the impact of shoe type on antiskid performance, the rough floor has a better anti-sliding effect [89].

For the HPt of D5, the various civil engineering experiments with complex process increase the probability of risk occurrence. For example, when conducting the relevant experiments of clayey soil, the operator’s hands will be stained with clayey soil. The operator might also ignore whether the experimental environment was made to be messy.

(2) The WF scores for D1, D2, D3, and D4 are the highest, as shown in Figure 4. In the WF, the impact of man, object, and management on the environment is relatively low because the environment is a relatively low-risk source compared to the others, which also coincides with the low LCI value of most environment calculated in Table 7. The score of D1′s S is higher, which may be due to the psychological pressure, fidgety, fatigue, and the decrease of experimental efficiency caused by strong noise vibration [90]. The noise produced by some experiments in civil engineering laboratory (such as grinding aggregate by ball grinder, crushing aggregate by jaw crusher and cutting concrete by saw) exceeds the limit value specified in the standard compared with chemical and biological experiments, etc. [91]. Hence, it is essential for civil engineering laboratories to strengthen noise management. This requires the laboratory to place sound-proof boards or sound-absorbing or sound-proof cotton and other materials in partition walls. The operators should wear hearing protection equipment when necessary [92], such as earplugs (such equipment should also be kept in the laboratory). For D3, the S and HPt are higher, which may be related to the toxic and harmful gases often produced by some civil engineering specialties (such as building environment specialty) when conducting the experiments. If the ventilation conditions are not good, the poisoning can easily occur. Ventilation is a key priority in laboratory design [50], but if the exhaust ventilation equipment is placed on the wall near the pollution source, it will effectively reduce the risk [48]. Other risk factors should be emphasized, e.g., for laboratories with poor temperature and humidity, air conditioning should be set in the laboratory. This can effectively improve the indoor temperature and humidity conditions, as well as the comfort of the operator [93]. Furthermore, improving the lighting in the learning environment can also inspire students to learn. Therefore, it is recommended to use the combination of sunlight and artificial light to provide lighting in the laboratory [51].

(3) From D1 to D5, the HD (including A, R, Se) and FD are relatively low. The relatively low level of HD maybe due to that the poor environment can be found by the operator (which can be identified), however whether the operator can take measures to change this dangerous environment depends on the operator himself.

## 7. Conclusions

Laboratories are important places for personnel training and scientific research in universities. They play an increasingly important role in universities, and its safety issues cannot be ignored. Although most of the existing research involve laboratory safety risks, little attention has been paid to civil engineering laboratories. The safety risks of civil engineering laboratories from the perspective of 13 risk factors were mainly discussed in four aspects of man, object, management, and environment. In the study, it is found that (1) the civil engineering laboratory is quite different from the common industrial laboratory, which has obvious specificities, and (2) 13 risk factors are more serious for civil engineering laboratories, among which A2 (insufficient safety awareness of operators), B2 (danger due to equipment failure), C1 (imperfect management policies), and D5 (complex floor conditions) are more worthy of closer attention. They need to be solved from the source. (3) At the same time, in each risk resource, worsening factors need to be analyzed and handled, especially the cross-influence among man, object, and management. (4) Finally, based on the analysis results, some targeted suggestions have been proposed.

The LCI model is applied to a new research field of civil engineering laboratories and the assessment indicators are systematically derived from four dimensions (man, object, management and environment) to create a risk list for civil engineering laboratories. We obtain LCI values of these risk factors and identify the highest priority. In this study, we revealed the current situation of civil engineering laboratories in Jiangsu Province and verified the feasibility of this method. Some parameters with lower scores have not been discussed in-depth in this research and thus need further study. The ideas and methods of this research can also be extended to the research of civil engineering laboratories, globally. Due to the restriction of research condition, we only take Jiangsu Province as an example in this paper. The current status and safety measures of civil engineering laboratories around the whole China and the difference in different regions are still waiting for further research. In the future, the safety risks in civil engineering laboratories in specific areas are expected to be evaluated or the method applied to the safety risk evaluation of a specific civil engineering laboratory. And more parameters of LCI, such as risk perception, should be considered for in-depth analysis in the future study.

## Figures and Tables

**Table 1:** Risk factors in civil engineering laboratories.

	Code	Risk Factors	Literature Sources	Risk Factors Description
Man	A1	Insufficient capacity of operators	Shariff A M (2012), Nouri J (2011), Adane L (2012)	Risk caused by misoperation, illegal operation, misjudgment of the experimental environment and weak emergency response ability
	A2	Insufficient safety awareness of operators	Walters A U C (2017), Alaimo P J (2010), Galante (2016)	Carelessness and a weak sense of responsibility
	A3	Poor physical and psychological conditions	Williamson A (2011), Das A K (2020)	Risk caused by over-fatigue, depression, and poor physical condition
Object	B1	Danger in the test product or mechanical equipment	Sims B (2005), Watson (2012)	Risk of fire and explosion (e.g., dangerous chemicals, electrical equipment, ignition sources, and electric circuits). High-temperature and high-pressure equipment may cause scald, impact, and other risks. Some equipment may cause mechanical injury and the like.
	B2	Danger due to equipment failure	Richards-Babb M (2010), Watson (2012), J. Meng (2004), National Research Council (2011)	Failure of safety valves, insufficient maintenance of instruments, leakages caused by the aging and insulation of equipment, unstable power supply, failure to monitor risk, and so on.
Management	C1	Imperfect management policies	Mulcahy M B (2017), Alaimo R J (2010), Stroud L M (2007)	Incomplete safety rules and regulations and inadequate safety supervision system resulting in the failure of timely maintenance of experimental equipment, incomplete safety education, or lack of necessary laboratory safety input, and so on.
	C2	Capital risk	Sengupta S K (2005)	Due to the lack of funds, the experimental equipment which has not been repaired is still in use or lacking necessary laboratory safety investment.
	C3	Weak execution of safety management	Grote G (2012)	The laboratory equipment is not maintained in time due to poor execution, or the necessary laboratory safety investment is lacking, there is corruption risk, or the laboratory safety education is not comprehensive, and so on
Environment	D1	Strong noise vibration	Kocsis D (2018), Griffiths P D (1970)	Strong noise vibrations can lead to psychological pressure on operators, fidgetiness, fatigue, and decreasing in experimental efficiency and mechanical equipment performance.
	D2	Adverse temperature and humidity	Chao P C (2013), Griffitt W (1970)	Poor temperature and humidity conditions will affect people’s psychophysiology and the performance of mechanical equipment.
	D3	Bad ventilation	Stroud L M (2007), Davardoost F (2019) Kerber S I N (2005), Secondo L E (2020)	Some chemical experiments of building environment and water supply and drainage specialty will use toxic and harmful gases. If they are not properly used and stored and the ventilation conditions are not effective, then they can easily cause poisoning.
	D4	Poor lighting	Samani S A (2012), Xu, X (2020)	If the environment is dark, people will have visual fatigue and reduced attention. If the illumination brightness is too high, it is difficult to clearly see the surrounding objects.
	D5	Complex floor conditions	Zhang Y (2009), Alaimo P J (2010), National Research Council (2011)	There are too many sundries, disorderly stacking, smooth ground, etc. in the workplace. The ground is relatively smooth and prone to fall accidents. If it collides with large machinery in a civil engineering laboratory, it will cause physical injury.

**Table 2:** Score of research specificities.

No.	Research Specificities	Code
1	The operators do not pay attention to the experiment and are not familiar with the experimental procedures due to free experimentation and indirect management of the laboratory leader.	rs_1_
2	Operators are not trained in normal risk identification and are not aware of possible risks.	rs_2_
3	Frequent replacement of operators and incomplete records in laboratory safety experience.	rs_3_
4	Operators and partners are not familiar with the possible risks of the experiment.	rs_4_
5	Operators are not very familiar with the laboratory environment, thus ignoring potential risks.	rs_5_
6	The complex and changeable experimental projects and a large number of students increase the difficulty of safety management.	rs_6_
7	The multicultural environment (such as the existence of foreign scholars) makes the operators have different cognition of experimental safety and risk factors, and there are communication barriers between operators.	rs_7_

**Table 3:** Explanations of lab criticity index (LCI) contributing factors.

Factors Names	LCI Factors	Factor Descriptions
Research specificities	RS	Research specificities are to distinguish civil engineering laboratories and industrial laboratories. The larger the value is, the higher possibility the factor is to make a difference between a civil engineering laboratory and an industrial laboratory.
Worsening factors	WF	The worsening factors present the influence among risk factors, in particular situations in which a risk can be worsened or enabled by the presence of another risk [60]. It can enlarge the direct consequence and severity of the risk. The larger the value is, the faster the danger will occur.
Severity	S	Severity refers to the degree of casualties, economic losses, or impact on the laboratory environment, school reputation, and the like. The larger the value is, the more serious the consequence will be.
Availability	A	Availability refers to whether most teachers and students who use the laboratory know the risk factors well. The larger the value is, the better operators know the risk factors.
Reliability	R	Reliability refers to whether the risk factors are easy to identify. The larger the value is, the easier it is for operators to identify the risk factors.
Selectivity	Se	Selectivity refers to whether the factor is easy to identify, even if there are multiple risk factors. The larger the value is, the easier it is for operators to identify the risk factors.
Hazard probability	HPt	Hazard probability of risk factors. The higher the value is, the easier it is for operators to identify the risk factors.
Frequency/Duration	FD	Frequency and Duration of students’ experiments. The higher the value is, the longer the total experiment time of students in the civil engineering laboratories.

Note: The LCI factors range from 2 to 10 [11].

**Table 4:** Descriptive statistical analysis of teachers’ basic information.

Option	Frequency	Ratio
Lecturers (who teach the courses with the requirements of experimental courses)	74	63.16%
Lab teachers	44	36.84%

**Table 5:** Descriptive statistical analysis of lecturers’ teaching specialty information.

Option	Frequency	Ratio
Civil Engineering (Construction Engineering)	24	32.43%
Traffic Bridge and Tunnel Engineering	15	20.27%
Geotechnical and Underground Engineering	11	14.86%
Civil Engineering Mechanics	9	12.16%
Civil Engineering Management	5	6.76%
Municipal Engineering	12	16.22%
Building Environment and Energy Application Engineering	8	10.81%
Others	12	16.22%

**Table 6:** Descriptive statistical analysis of teachers’ lab work experience information.

Years of Lab Work Experience	Frequency	Ratio
Less than 5 years	17	14.41%
5 years or more	101	85.59%

**Table 7:** LCI factors results.

Risk Factors		LCI Factors							LCI
		WF	S	A	R	Se	HPt	FD	
Man	A1	7.254	6.305	4.966	4.746	5	5.288	5.610	6.984
	A2	6.932	6.712	4.61	4.763	4.729	5.695	5.610	7.097
	A3	7.367	6.203	4.186	4.644	4.661	5.678	5.610	6.921
Object	B1	7.079	6.203	5.068	4.949	5	5.966	5.610	7.006
	B2	7.362	6.424	5.051	5.508	5.542	5.763	5.610	7.142
Management	C1	6.977	6.305	5.441	5.39	5.576	5.627	5.610	7.047
	C2	6.158	6.22	5.729	5.424	5.203	5.559	5.610	6.907
	C3	6.881	5.949	5.492	5.085	4.881	5.814	5.610	6.855
Environment	D1	7.102	6.017	4.661	5.102	4.881	5.288	5.610	6.826
	D2	6.994	5.475	4.644	5	4.983	5.271	5.610	6.642
	D3	7.192	5.932	4.661	4.915	4.949	5.814	5.610	6.864
	D4	7.203	5.525	4.881	4.881	4.898	5.203	5.610	6.739
	D5	7.249	6.576	4.898	5.136	5.153	6.22	5.610	7.182

**Table 8:** Scores of worsening factors.

Factors		Worsening Factors				Mean Value
		Man	Object	Management	Environment	
Man	A1		7.983	7.525	6.254	7.254
	A2		7.068	7.441	6.288	6.932
	A3		7.373	7.373	7.356	7.367
Object	B1	7.373		7.373	6.492	7.079
	B2	7.542		7.763	6.780	7.362
Management	C1	7.203	7.271		6.458	6.977
	C2	6.169	6.407		5.898	6.158
	C3	6.915	7.017		6.712	6.881
Environment	D1	7.051	7.254	7.000		7.102
	D2	6.864	7.000	7.119		6.994
	D3	7.220	7.136	7.220		7.192
	D4	7.102	7.271	7.237		7.203
	D5	7.407	7.034	7.305		7.249

**Table 9:** Scores of research specificities.

Research Specificities	rs_1_	rs_2_	rs_3_	rs_4_	rs_5_	rs_6_	rs_7_	Total
Original Data	3.500	3.610	3.737	3.788	3.703	3.695	3.500	
Score (=Original Data /3.5)	1.000	1.031	1.068	1.082	1.058	1.056	1.000	7.295

## Supplementary Materials

The following are available online at https://www.mdpi.com/1660-4601/17/17/6244/s1, Table S1: Original data of questionnaire.
